# Animal models of rheumatoid pain: experimental systems and insights

**DOI:** 10.1186/s13075-017-1361-6

**Published:** 2017-06-30

**Authors:** Bradford D. Fischer, Adeshina Adeyemo, Michael E. O’Leary, Andrea Bottaro

**Affiliations:** Department of Biomedical Sciences, Cooper Medical School of Rowan University, 401 S. Broadway, Camden, NJ 08103 USA

**Keywords:** Arthritis, Inflammation, Pain, Animal models, Nociception

## Abstract

Severe chronic pain is one of the hallmarks and most debilitating manifestations of inflammatory arthritis. It represents a significant problem in the clinical management of patients with common chronic inflammatory joint conditions such as rheumatoid arthritis, psoriatic arthritis and spondyloarthropathies. The functional links between peripheral inflammatory signals and the establishment of the neuroadaptive mechanisms acting in nociceptors and in the central nervous system in the establishment of chronic and neuropathic pain are still poorly understood, representing an area of intense study and translational priority. Several well-established inducible and spontaneous animal models are available to study the onset, progression and chronicization of inflammatory joint disease, and have been instrumental in elucidating its immunopathogenesis. However, quantitative assessment of pain in animal models is technically and conceptually challenging, and it is only in recent years that inflammatory arthritis models have begun to be utilized systematically in experimental pain studies using behavioral and neurophysiological approaches to characterize acute and chronic pain stages. This article aims primarily to provide clinical and experimental rheumatologists with an overview of current animal models of arthritis pain, and to summarize emerging findings, challenges and unanswered questions in the field.

## Background

### Arthritis pain in human patients

Rheumatoid arthritis (RA) and spondyloarthritis are prevalent inflammatory-erosive joint diseases which affect as many as 2% of the population worldwide, causing severe, debilitating morbidity and major economic costs due to both health care expenditures and lost productivity. Inflammatory arthritides are characterized by progressive joint inflammation and destruction, deformity, loss of mobility, systemic manifestations and severe pain which ultimately hamper basic motility functions, activities of daily living and psychological health in the affected individuals [1, 2].

Therapeutic approaches focused on the underlying inflammatory immunopathology have led to the introduction of targeted biological disease-modifying anti-rheumatic drugs (DMARDs), pioneered by anti-tumor necrosis factor (TNF) agents, which have revolutionized the clinical treatment and dramatically improved long-term outcomes of these diseases [3, 4].

Pain, initially joint localized but often progressing to widespread in advanced stages, is a major component of inflammatory arthritis symptomatology and is typically the primary reason for initial rheumatological referrals [1, 2]. In a subset of patients with advanced disease, chronic pain can also acquire typical neuropathic features [1, 5]. These and other clinical findings support a key role of neurosensitization mechanisms of nociceptive pathways in the central nervous system in the establishment of chronic arthritic pain.

The therapeutic success of biologic DMARDs has provided new insights into the unique qualities of pain manifestations associated with chronic inflammatory arthritis. Notably, patients with a positive response to anti-TNF agents often report rapid initial therapeutic pain suppression which precedes the clinical anti-inflammatory response [1, 6]. This is consistent with a direct role of TNF and other inflammatory cytokines like IL-6 and IL-17 on nociceptor sensitization pathways [7]. Indeed, nociceptors in dorsal root ganglia have been shown to express TNF receptors and to directly respond to TNF stimulation [7].

More problematic for the clinical management of these diseases, however, is that a significant fraction of patients fail to report long-term suppression of pain comparable to their anti-inflammatory clinical response to biologic and other DMARDs [1, 8–10]. Based on these clinical observations and treatment outcomes, therefore, direct inflammatory pain pathways are generally thought to predominate in early-stage arthritis, evolving into chronic-neurogenic pain mechanisms over time [1, 5].

From the patients’ perspective, effective management of arthritis-associated pain is a primary therapeutic goal, and a major component of patient-driven disease assessment often underestimated by clinical disease activity scoring tools [2, 10, 11]. A fuller mechanistic understanding of the inflammatory and neuroadaptive mechanisms leading to chronic arthritic pain is therefore required to address a major unmet need in patient care.

This review will first describe existing animal models of arthritis that are commonly utilized for preclinical pain studies, discussing their specific advantages and drawbacks. We will then provide key background on experimental methods for quantitative assessment of pain responses in animal models, highlighting important theoretical and practical challenges, and summarizing recent insights into the mechanisms of arthritic pain.

### Animal models of inflammatory joint disease

Animal disease models have proven invaluable to unravel the pathophysiological pathways of inflammatory arthritis, and for investigational testing of therapeutic agents. The most commonly utilized animal species for this purpose are mice and rats, either as strains that spontaneously develop arthritis or as inducible models in which disease can be provoked by administration of arthritogenic stimuli. A number of comprehensive reviews have already covered the range of animal arthritis models, and their features in comparison to human disease pathophysiology and therapeutic responses [12–15]. Here, we will briefly summarize key aspects of a few relevant models, particularly with respect to features such as disease onset, progression and chronicity which may affect their use in pain studies.

#### Spontaneous arthritis models

Several rodent strains have been reported to be susceptible to development of spontaneous arthritis, but experimental studies have primarily focused on a few genetically modified mouse strains which display full penetrance and reproducible disease progression, especially K/BxN and TNF-transgenic (TNFtg) mice [16, 17].

##### The K/BxN model

K/BxN mice express a T-cell receptor transgene specific for a peptide derived from the ubiquitous enzyme glucose-6-phosphate isomerase (GPI), presented by the I-Ag^7^ MHC-II allele [17, 18]. Autoimmunity manifests with onset of joint inflammation around 3–4 weeks of age, progressing over 4–8 weeks to full inflammatory-erosive arthritis. Anti-GPI autoantibodies appear to be the primary drivers of disease, because the transfer of K/BxN serum, or even K/BxN-derived anti-GPI monoclonal antibodies, is sufficient to induce arthritis in other mouse strains (see later) [19]. Histologically, K/BxN disease closely parallels findings in human RA joints, including pannus formation, inflammatory infiltrates and articular erosions. Therefore, K/BxN mice replicate human RA both in the autoimmune pathophysiology and key disease features.

##### The TNF-transgenic mouse model

TNFtg mice, derived in the early 1990s, express a human TNF gene lacking post-transcriptional regulatory elements, and have provided cornerstone evidence for the involvement of TNF in inflammatory arthritis [16]. Commonly used strains range from a single copy (Tg(TNF)3647 strain) to multiple copies (Tg(TNF)197 strain and others) of the TNF transgene [16, 20]. Other TNF-overexpressing strains with similar disease features were later developed, but will not be discussed here.

The 3647-strain TNFtg mice display delayed onset of joint inflammation compared to multicopy transgenics (6–8 weeks of age vs 3–4 weeks), slower disease progression (12–16 weeks from onset to maximal severity) and increased lifespans (over a year with appropriate husbandry) [16, 20]. Because of their late onset and slow progression, single-copy TNFtg mice are particularly suited for the study of processes associated with preclinical disease stages and with progressing chronicity. Although TNFtg disease is not autoimmunity driven, it displays many of the histopathological findings of human RA (synovial hyperplasia, neutrophilic inflammatory infiltrates and joint erosion) and other signs of systemic inflammation. TNFtg mice are therefore an excellent model to investigate TNF-induced inflammatory pathways in human disease.

#### Inducible arthritis models

Inflammatory arthritis can be induced experimentally in many species, with well-established systems utilizing both rats and mice. Arthritogenic signals in these models can consist of nonspecific inflammatory agents, such as different types of adjuvants; of immunization procedures using specific antigens which cause self-tolerance breakdown; or of passively administered autoreactive antibodies or sera. Compared to spontaneous disease strains, advantages of inducible models include their cost-effectiveness, reduced husbandry needs and reproducibility of existing protocols. Disease typically develops rapidly, limiting the physiological windows for the study of disease onset and progression. Penetrance, persistence and chronicity of arthritis vary depending upon the model.

##### Adjuvant-induced arthritis and related models

Arthritis can be reproducibly induced in susceptible strains of rats (e.g., Lewis or DA rats) by intradermal injection of adjuvants, including complete or incomplete Freund’s adjuvant (CFA, IFA), pristane and squalene, or intraarticular administration of streptococcal cell wall products or antigens in presensitized rats or mice [13, 21]. In most applications, disease follows within days of administration, reaches maximal severity within one to a few weeks and is typically followed by remission, which in some protocols is reactivatable by repeated treatments. Disease intensity and course vary depending on the strain and arthritogenic signal. For instance, CFA-induced arthritis is significantly more severe and systemic than that induced by antigen-free adjuvants like IFA or pristane. Pristane-induced arthritis in rats displays a remitting–relapsing “flaring” pattern that resembles human RA. Although the inciting stimulus in adjuvant-induced arthritis is not antigenic, the resulting disease is often associated with MHC-linked susceptibility, production of autoantibodies and/or emergence of autoreactive T-cell clones, reflecting an autoimmune pathophysiology [13].

##### Collagen-induced arthritis

Collagen-induced arthritis (CIA) is the most frequently utilized experimental model of arthritis. Inflammatory arthritis is induced in genetically susceptible rats, mice, rabbits and other species by immunization with type II collagen, typically of bovine origin [22]. In a typical mouse protocol, polyarthritis develops a few weeks post immunization with CFA-emulsified collagen, reaching maximal clinical severity within 2 weeks of onset and persisting in a chronic state thereafter.

##### Antibody-induced arthritis

Evidence that serum from collagen-immunized rats and mice could passively induce arthritis in recipients provided early experimental confirmation of the direct pathogenic role of humoral immunity in arthritis. The same approach is commonly utilized to induce acute, transient arthritis in rats or mice, in models such as collagen-antibody-induced arthritis (CAIA) and K/BxN serum-transfer arthritis [19, 23]. Arthritis in these models is mediated by immune complex deposition in the joints, recruitment of neutrophils and other inflammatory innate components, and is independent of B and T cells [19, 23]. Histologically, bone and cartilage erosions and pannus formation closely resemble human RA. Because the inflammatory response in the joints is rapid, reproducible and intense, the models are best suited for the study of acute mechanisms.

CAIA can be induced in either rats or mice by intravenous transfer of a mixture of anti-collagen II monoclonal antibodies, most often accompanied by intraperitoneal injection of LPS to potentiate the effect. Disease onset follows rapidly after LPS injection, reaching maximal severity in 4–5 days and waning in about an additional week. Repeated injections can exacerbate and extend the response.

Similarly, transfer of serum, antibodies or anti-GPI monoclonals from K/BxN mice (see earlier) can induce rapid (2–3 days) onset of polyarthritis in recipients, with almost complete penetrance and without additional inducers [19].

### Experimental systems for analysis of pain-associated responses in animal models

Self-reported pain scores are the primary means for evaluating pain severity in patients. However, objective assessment of pain in animals represents a significant challenge for preclinical research, which has led to the development of several experimental systems which reproducibly mimic pathological pain conditions in humans, including inflammatory and neuropathic pain, and allow assessment of their outcomes. Broadly speaking, animal behavioral models of pain consist of two principal components: experimental manipulation intended to produce a pain-like state; and measurement of behavior presumably indicative of that pain state. These models can be used to experimentally assess pain as well as its relief following the administration of antinociceptive drugs.

Current methods of pain assessment in animals were initially developed in the context of models of induced acute pain from different types of noxious stimuli, or of neuropathic pain following experimental nerve injury, but the same approaches are routinely applied to arthritis pain models. All of these approaches have to overcome the major conceptual hurdle of translating the subjective experience of pain in animal subjects into investigator-observable, quantifiable responses.

Broadly, experimental assessment of pain in animal models relies on the quantification of pain-evoked or pain-suppressed behaviors. Pain-evoked behaviors may occur at very low rates in the absence of pain, and increase in frequency following putative nociceptive stimuli. Examples include footpad withdrawal, jumping, flinching or licking. In contrast, pain-suppressed behaviors are those that occur at high rates in the absence of noxious stimuli and decrease in magnitude or duration after exposure to a noxious stimulus. Some natural behaviors that may be suppressed in this context include locomotor activity, nesting, motor coordination/balance or feeding (e.g., [24]). In each case, measurable changes in behavior may result from pain responses to a normally noxious stimulus, an enhanced response to low-grade painful stimuli (hyperalgesia), or a painful response to a normally non-noxious stimulus (allodynia).

#### Types of experimental pain models

The basic concept of measuring acute nociceptive pain in animals relies on the input of a normally noxious stimulus followed by the assessment of a withdrawal response. The noxious stimulus can vary in intensity and in modality, such as electrical, mechanical, thermal or chemical [25, 26].

The advantage of these models largely rests in their simplicity, their ability to objectively measure the withdrawal response and their predictive value to assess pharmacological effectiveness of opioid analgesics in humans [25]. However, they have limited clinical relevance and show impaired validity when nonopioid analgesics are tested, such as steroids and nonsteroidal anti-inflammatory drugs (NSAIDs). These and other limitations have led to the development of additional methods to behaviorally assess more clinically relevant pain states, including models of acute and chronic inflammation and neuropathy.

Classic models of acute inflammatory pain include the injection into the hind paw of rodents of chemicals (e.g., formalin or carrageenan), which produce a rapid nociceptive response characterized by paw flinching and licking (formalin), and decreased response thresholds to thermal and mechanical stimuli (i.e., allodynia and hyperalgesia). Neuropathic pain in animals is classically modeled by interventions that cause some degree of nerve injury, such as ligation or chronic constriction. While these systems have good predictive validity in pharmacological studies (e.g., sensitivity to opioids and anticonvulsants), they do not replicate the etiology of most human neuropathic states, nor some of their common clinical manifestations [27, 28].

These experimental systems have been instrumental to unraveling basic neurophysiological pathways of nociception and for pharmacological research, but because of their limited clinical applicability they have been progressively complemented by more pathologically relevant models of chronic and/or neuropathic pain, including the inflammatory arthritis models already described.

#### Quantifying the perception of pain in animals

The assessment of pain in animals is naturally fraught with conceptual and experimental complexities, and significant research has been carried out in recent years to standardize protocols, identify the impact of critical variables (such as sex, age, behavioral and environmental factors), reduce investigator-associated subjectivity and disruption, and expand the range of testable responses from strictly sensory to include psychoaffective components [26, 29–31]. A summary of commonly utilized methods is presented in Table 1.

**Table 1:** Experimental methods of pain assessment in rodent arthritis models

Assessment method [example references]	Response measured	Pain aspect assessed	Advantages	Disadvantages
Von Frey test/mechanical hyperalgesia [6, 32, 33, 48–54, 56, 58, 59, 62]	Pain-evoked behavior: withdrawal threshold from a mechanical stimulus	Mechanical allodynia/hyperalgesia	Quantitative, well-established protocols	Stimulation of mechanical and nociceptive fibers; possible investigator bias/subjectivity
Hargreaves test/thermonociception [6, 33, 51, 53, 54, 56–59, 62]	Pain-evoked behavior: withdrawal latency from a thermal stimulus	Thermal allodynia/hyperalgesia	Quantitative, well-established protocols; primary stimulation of nociceptive fibers	Possible investigator bias/subjectivity
Ambulatory/locomotor behavior [53, 56, 59, 61–66]	Pain-suppressed behavior: locomotion in an open field	Locomotor activity/ambulation/exploratory behavior	Automated quantitative measurement; may include affective component	May be affected by nonpain-related outcomes (e.g., motor function)
Grimace scales [38]	Changes in facial expressions associated with pain	Expression of subjective pain perception	Non-interventional; directly linked to individual pain state; may include affective component	Possible investigator bias/subjectivity; experimenter training needed; further validation in arthritis models required
fMRI [6]	Functional changes in CNS activity associated with pain	Affective CNS responses to pain	Objective measurements; may include affective component	Expensive equipment; high-level investigator training needed; requirement for restraint/sedation
Gait/dynamic weight bearing analysis [34, 59]	Changes in ambulatory posture or weight distribution	Spontaneous gait changes due to joint pain	Objective, quantitative measurements; automated systems available	Specialized equipment needed.
Operant conditioning [60]	Behavior emitted to receive a reward despite concurrent exposure to a painful stimulus	Affective and/or motivational components of pain perception	Objective, quantitative; automated systems available; may include affective component	Specialized equipment needed
Escape/avoidance [45]	Latency to escape noxious stimulus	Affective and/or motivational components of pain perception	Objective, quantitative; automated systems available; may include affective component	Specialized equipment needed

*CNS* central nervous system, *fMRI* functional magnetic resonance imaging

##### Pain-evoked behaviors

The most frequently utilized techniques for assessment of ongoing pain in experimental animals rely on development of hyperalgesia and allodynia, which can be quantitatively measured by assessing withdrawal from non-noxious or subthreshold stimuli. Arguably the most common mechanical stimuli used for this purpose in rodents are von Frey hairs, elastic filaments of varying diameter that buckle at a defined force. Applied to the plantar surface of the paw when the animal is positioned over a wire mesh surface, von Frey hairs of increasing stiffness allow the determination of the mechanical threshold of paw withdrawal. Although less frequently utilized, electrical stimuli can also be applied briefly and quantifiably, and are reproduced easily.

Lowered thresholds of evoked responses to stimuli relative to controls are considered hallmarks of allodynia and hyperalgesia. One limitation of these approaches lies in the subjective measurement of withdrawal responses, which can be obviated by use of automated electronic systems [32, 33]. Assays based on evaluation of weight bearing may be able to more objectively and physiologically identify postural changes in models of joint inflammation [34].

Neurophysiologically, the primary caveat of mechanical and electrical stimuli is that they activate both low-threshold mechanoreceptors and nociceptors, preventing a clear-cut distinction of the pathways involved. Thermal stimulation is thought to be more specific in directly activating nociceptive fibers. Commonly used methods involve applying radiant heat or immersing the distal end of the tail of a restrained animal into a thermostatic water bath. Unrestrained animals can be tested using Hargreaves’ method, during which radiant heat is applied to the plantar surface of the footpad via an infrared source to elicit paw withdrawal.

##### Functional magnetic resonance imaging

Functional magnetic resonance imaging (fMRI) measures changes in paramagnetic signals secondary to oxygen extraction from oxyhemoglobin, reflecting metabolic activity in brain tissue. Human fMRI studies have demonstrated activation in specific brain structures following noxious stimuli, including in the lateral thalamus, primary and somatosensory cortex, insular cortex, anterior cingulate cortex, striatum, cerebellum, supplemental motor area and periaqueductal gray matter [35]. Small animal neuroimaging studies have validated the use of fMRI to study pain in animals, showing activation in similar regions [6, 36]. fMRI has the distinct advantage of being able to assess pain-related effects in brain areas thought to be important in processing both sensory and affective components. However, the approach requires trained personnel and sophisticated equipment, and the use of restraint or sedation to minimize head movement during data acquisition adds obvious confounding variables.

##### Grimace scales

Recent studies have analyzed facial expressions in animals in response to painful stimuli. A 10-point facial expression (“grimace”) scale was developed in mice based on orbital tightening (closed eyelid or eye squeeze), nose bulge, cheek bulge, ear position and whisker changes following intraperitoneal administration of acetic acid [37]. Similar scales exist for rats and rabbits, and are being evaluated for other species. Of note, analogous facial expressions are exhibited by humans that verbally report pain, and can be utilized to assess pain responses in nonverbal humans. Grimace scales have shown good reproducibility for trained investigators, especially in acute pain models, and have the distinct advantage of allowing direct assessment of pain in disease models in the absence of additional experimental interventions. However, their applicability to joint inflammation and chronic pain states remains to be fully validated [38].

##### Pain-suppressed behaviors

Although pain-evoked behaviors are most commonly utilized in animal pain studies, pain-suppressed behaviors have also been used to assess pain in animals. These are defined by a decrease of otherwise healthy behaviors that occur at high rates (e.g., feeding, spontaneous ambulatory behavior) following exposure to a noxious stimulus. These have clinical correlates in human chronic pain patients, where suppressed behaviors may include decreased activities of daily living or ambulation, and correlate with signs of clinical depression. Evidence of pain-suppressed behaviors in animals may be quantified by reduced feeding, reduced mating and/or reduced locomotor activity.

**Fig. 1:**
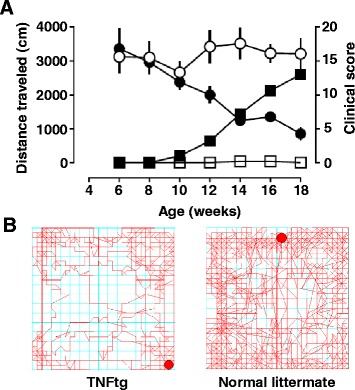
Correlation between locomotor activity and clinical disease progression in TNFtg mice. **a** Ambulatory behavior, measured as distance traveled in 60-minute experimental sessions (*left*, *circles*) was assessed in female TNFtg mice (*n* = 5, *filled symbols*) and normal littermates (*n* = 6, *open symbols*) every 2 weeks starting at 6 weeks of age. At the same times, disease progression was assessed by traditional clinical scoring of joint inflammation in each paw, on a scale from 0 to 4/paw (maximum score = 16) (*right*, *squares*). Locomotor activity was quantified in an open field arena (27.3 cm × 27.3 cm × 20.3 cm) equipped with a computer interface and software (MED Associates, St. Albans, VT, USA) and a 16-beam infrared array positioned along the *X* and *Y* axes of the enclosure. Symbols represent means and SEM for each data point. Note how the increases in clinical scores in TNFtg mice (*filled squares*) parallel the decline in their locomotor activity (*filled circles*) over time. **b** Example of recorded locomotor tracings from a TNFtg mouse (*left*) and a WT littermate (*right*) at 16 weeks. Note the reduction in locomotor/exploratory behavior in the TNFtg mouse. *TNFtg* TNF-transgenic

Decreased locomotor activity has been associated with pain-like states in animals, including in rodent models of inflammatory and neuropathic pain, although with some discrepancies [39, 40]. As an example, Fig. 1 shows the close quantitative correlation of decreased locomotor activity in TNFtg mice with traditional clinical scores of joint inflammation during disease progression. Models of pain suppressed behaviors such as these can be used to preclinically model the decreased activity observed in patients with RA.

The inclusion of pain-suppressed behaviors in animal testing has several advantages. First, pain-suppressed behaviors can be objectively measured using automated equipment (e.g., locomotor activity box, operant response chambers). Second, drugs that produce motor impairment are less likely to produce false positive effects in pain-suppressed assays relative to pain-stimulated behaviors. Third, measures of pain-suppressed behaviors may be used to investigate some of the psychoaffective components of pain and may improve the translational validity of these behaviors toward the clinic. At the same time, the complexities of interpreting animal behavior from a psychoaffective standpoint cannot be understated, and important work needs to be carried out in this area to fully validate these approaches [29, 30].

##### Operant conditioning and behavioral avoidance

Similar to study of pain-suppressed behaviors, behavioral methods can be utilized to explore affective and/or motivational changes that occur in response to pain, bypassing some of the problems associated with pain-evoked responses. Operant procedures may require an animal to predictably emit a defined behavioral response, such as traversing a noxious stimulus (e.g., pass through a heat source), in order to obtain in a positive reinforcer (e.g., food treat) [41, 42]. Place avoidance or preference paradigms are based on the assumption that animals will display aversive behavior toward noxious stimuli (e.g., avoid heated cage areas) or preference for environments associated with reward [43–45]. Changes in avoidance behavior, such as in the presence of persistent pain states or after experimental manipulations (e.g., analgesic drugs), are thought to be related to changes in nociceptive pathways. Assays such as these can be used to study processes that are thought to involve higher brain centers relative to peripheral nociceptors.

### Recent advances in animal models of arthritic pain

Although animal models of arthritis have been widely utilized for decades to study not just disease pathogenesis and candidate therapeutics, only in the past 10–15 years have the biological properties of pain in these systems begun to be investigated systematically [46, 47].

#### Links between inflammation and pain-evoked responses

In all models studied, inflammatory disease is associated with lowered thresholds to mechanical and thermal stimulation, reflecting hyperalgesia and allodynia. However, thermal and mechanical hypersensitivity are not always closely correlated to each other, suggesting that strain-specific and method-specific differences should be considered in evaluating experimental outcomes [33]. In addition, age may be an additional variable, because adjuvant-induced arthritis evoked similar inflammatory responses in young and old mice, but induced higher levels of mechanical hypersensitivity in younger mice using the von Frey test [48]. These discordances aside, sensitization of pain pathways is typically concomitant with the appearance of clinical signs of inflammation, and in some cases it can precede them [46, 49]. This is consistent with pain often being the earliest disease manifestation in human RA patients [1, 50].

Studies focused on the resolution end of the disease spectrum using transient antibody-induced arthritis (e.g., CAIA and K/BxN serum transfer) have shown that pain sensitization can persist for extended periods of time beyond the resolution of inflammation [49, 51, 52]. This also parallels the discordance between the therapeutic control of inflammatory disease and persisting pain experienced by some human RA patients [2, 8–10]. Interestingly, transfer into mice of anti-citrullinated peptide antibodies from human RA patients was recently shown to evoke pain-like induced and suppressed behaviors in the absence of a detectable inflammatory response, suggesting that some pathogenic antibodies may mediate nociceptive signals by distinct, non-inflammatory mechanisms [53].

#### Neurophysiology of inflammatory pain

Molecular and cellular studies of nociceptors and non-neuronal cell types in dorsal root ganglia (DRG) and spinal cord sensory pathways have begun to elucidate the neurophysiological mechanisms of hyperalgesia in models of arthritis and other inflammatory diseases [32, 52, 54–56]. As in human patients, evidence is accumulating that arthritis chronicity in animal models is associated not just with nociceptor sensitization, but also with bona-fide neuropathic changes, as highlighted by upregulated expression of the neuronal transcription factor ATF3 and other stress markers in DRGs of long-term arthritis models [33, 51, 52, 56, 57]. In the K/BxN serum transfer model, the transition from acute to chronic pain states was shown to be associated with Toll-like receptor 4 function [58], making this molecule and its potential endogenous ligands intriguing therapeutic targets.

#### Analgesia in chronic arthritis models

Pharmacologically, consistent with a neurogenic mechanism, persistent pain in both CAIA and K/BxN mice appears to be alleviated by gabapentin, but not NSAIDs [49, 51]. A role for leptin-dependent opioid reward mechanisms and analgesia has been identified recently in rat adjuvant-induced arthritis, potentially expanding the usefulness of these models to human addiction studies [45]. TNF antagonists decreased pain responses, as assessed by locomotor/behavioral test and mechanical and thermal hyperalgesia, more rapidly than their anti-inflammatory activity in a model of rat antigen-induced monoarthritis and in TNFtg mice [6, 59]. The latter results correlated with fMRI findings in both animals and human patients, suggesting centrally mediated pain modulation by TNF [6].

#### Pain-suppressed behavior in arthritis models

Various forms of pain-suppressed spontaneous behaviors, including locomotor activity, as well as operant responses and place avoidance have also been studied in arthritis models, showing strong correlation with clinical disease [45, 60–66]. While the kinetics of clinical disease and suppression of locomotion appear to match closely in rapid-onset models of arthritis [61, 63, 65], the more slowly progressing K/BxN mouse strain displays a significant delay between peak clinical progression and decreased mobility [64]. This finding is suggestive of a psychoaffective component to the pain-suppressed behaviors in this strain. The possibility of depressive-like behavior resulting from chronic inflammatory arthritis was specifically investigated in TNFtg animals; however, the study failed to identify neurobiological or behavioral correlates of depression [66]. Whether these negative results reflect strain-dependent or experimental system differences or can be generalized remains to be established, but this is of course a crucial line of research due to the known psychoaffective component of pain in human RA patients [1, 2].

## Conclusions

Although no animal model perfectly recapitulates all aspects of human inflammatory arthritis, the diversity of existing models provides a large armamentarium for elucidating specific pathophysiological mechanisms, including the study of arthritic pain. In this respect, important criteria for model selection include relevant pathophysiology and disease kinetics, especially with regard to chronicity. Similarly, the array of systems currently utilized for the experimental evaluation of pain perception in animals—spanning from traditional quantitation of hyperalgesia and allodynia-linked responses, to behavioral studies, to the more recent neuroimaging and neurobiological approaches—offers important experimental opportunities, while also requiring thoughtful consideration of technical and interpretative caveats. Parallel progress in these two fields will greatly broaden our understanding of pain mechanisms beyond what can be achieved in human studies.

Key priority targets for this research effort include the mechanisms that establish pain chronicity and the emergence of neuropathy in inflammatory arthritis, which are critical for treatment and prevention of symptom progression. Despite the recent pharmacotherapeutic advances, pain remains a major unresolved need in the management of arthritis patients. Animal models have proven instrumental in providing key insights into the inflammatory pathophysiology of arthritis, leading to the biological therapeutics revolution. Application of the same preclinical approaches has great potential for the replication of this success in treating rheumatoid pain.
